# Synergistic effect of tea epigallocatechin gallate and tea flower-derived oligosaccharides on frozen storage cycles inducing oxidation and microstructural alterations of snakehead surimi proteins

**DOI:** 10.1016/j.fochx.2026.103518

**Published:** 2026-01-09

**Authors:** Noman Walayat, Shufan Ren, Piotr Kulawik, Asad Nawaz, Isam A. Mohamed Ahmed, Moneera O. Aljobair, Ibraheim Khalifa, Zhucheng Su, Ran Wei, Suleiman A. Althawab

**Affiliations:** a College of Tea Science and Tea Culture, Zhejiang Agriculture and Forestry University, 311400 Hangzhou, China; b Department of Animal Products Processing, University of Agriculture, Balicka 122, 30-149 Kraków, Poland; c College of Chemistry and Bioengineering, Hunan University of Science and Engineering, 425199 Yongzhou, Hunan, China; d Department of Food Science and Nutrition, College of Food and Agricultural Sciences, King Saud University, P. O. Box 2460, Riyadh 11451, Saudi Arabia; e Department of Sports Health, College of Sports Sciences and Physical Activity, Princess Nourah bint Abdulrahman University, Riyadh, Saudi Arabia; f Food Technology Department, Faculty of Agriculture, Benha University, Moshtohor, Qalyubia, Egypt

**Keywords:** Tea flower oligosaccharide, Oxidation, Surimi, Frozen storage, Proteins

## Abstract

The synergistic effect of epigallocatechin gallate (EGCG) and tea flower oligosaccharides (TFO) was analyzed in snakehead during different 8 frozen storage cycles (FCY). Four different protein groups were prepared: C (no additives), K (conventional combination (4% sucrose+4% sorbitol)), EGCG-TFO (1%), and EGCG-TFO (3%). FCY accelerated the oxidation, especially C, where decreases in total sulfhydryl (54.68 to 34.70 mmol/kg) and Ca^2+^ATPase (0.518 to 0.304 mmol/g) were observed, as well as an increase in surface hydrophobicity. The K samples showed better stability against FCY-induced oxidative modifications than EGCG-TFO (1%). Meanwhile, EGCG-TFO (3%) restricted the FCY accelerated increase in carbonyls from 1.56 to 5.17 nmol/mg. Moreover, the Amide *I* and II regions were preserved by promoting hydrogen and ionic interactions, as well as securing the α-helix in the myosin region, as evidenced by molecular simulation. EGCG-TFO (3%) also protected the microstructural attributes owing to its inhibitory role in protein coagulation and interaction forces.

## Highlights

• The EGCG-TFP mixture was assessed on snakehead protein during FCY.
• The EGCG-TFP mitigated the oxidation promoted decline in Ca2 + ATPase and carbonyls.
• The EGCG-TFP significantly maintained the Amide I and II associated alterations.
• The EGCG-TFP maintained the microstructural attributes of surimi muscles.
• Overall, EGCG-TFP (3%) was effective in preserving the snakehead quality.

## 1 Introduction

Snakehead fish (*Channa striata*) is a commercially significant species in global aquaculture, known for its robust nutritional profile, rapid growth rate and desirable culinary attributes, particularly in Asian markets (Yan et al., 2024). Its muscle proteins exhibit excellent gel-forming capabilities, making them highly suitable for producing surimi-based products, such as sausages or meatballs (Huang et al., 2023). These products are rich sources of concentrated proteins but are inherently susceptible to oxidative damage, denaturation and aggregation during processing and storage (Chen et al., 2024). However, fluctuating and extended frozen storage often increases protein oxidation, denaturation and aggregation, which in turn damages the protein microstructure, ultimately compromising the quality of the product (Sharma, Majumdar, Mehta, Ngasotter, & Gaurav, 2024; Sharma, Majumdar, Mehta, Panda, & Ngasotter, 2024). Furthermore, temperature fluctuations inherent to transportation, retail display and domestic storage exacerbate protein degradation, posing a persistent challenge to the maintenance of surimi and related meat product integrity. They also promote ice crystallization and recrystallization within the protein matrix, driven by free water migration, causing significant microstructural damage to surimi proteins (Tian et al., 2022). This damage is crucial to surimi gel formation, nutrition and overall quality during frozen storage or supply chain.

To mitigate these effects, the surimi industry typically relies on commercial cryoprotectants, such as sucrose, sorbitol, trehalose and phosphate (Tian et al., 2022). Although cost-effective and readily-available, these additives have limitations, including suboptimal cryoprotective efficacy against freeze-induced oxidative damage. Moreover, their high caloric content and sweetness raise consumer health concerns, driving the search for natural and effective alternatives (Nakazawa & Okazaki, 2020). Consequently, identifying abundant natural compounds capable of stabilizing proteins during frozen storage and under variable supply chain conditions is a research priority.

Epigallocatechin gallate (EGCG) is a tea-derived catechin, well known for its potential antioxidative properties. The availability of multiple hydroxyl groups (-OH), gallate moiety and polyphenolic backbone makes it a potential cryoprotectant candidate (Xiao et al., 2024). Moreover, as a potential bioactive compound, EGCG contains hydroxyl groups, which make it an effective antioxidant. These groups offer hydrogen atoms to neutralize free radicals, thereby preventing protein oxidation during extended frozen surimi storage. In addition to its antioxidative role, EGCG is also an excellent source for establishing hydrophobic interactions and hydrogen bonds, as well as cross-linking with amino acid functional sites (Zhou et al., 2024). Tea flowers (*Camellia sinensis*), a rich source of bioactive compounds, yield tea flower oligosaccharides (TFO) with significant potential for various applications. These non-starch, protein-bound acidic compounds consist primarily of neutral sugars (≈44.2%) and uronic acids (≈43.1%), with a minor protein component (≈3.5%) (Hu et al., 2022). Their carbohydrate profiles include galactose, rhamnose, arabinose, glucose, ribose, xylose, mannose and glucuronic acid (Hu et al., 2021). Naturally, the TFOs are hetero-oligosaccharides that aid the formation of extensive hydrogen bonds with protein and water molecules. These attributes assist in forming the hydration shell of myofibrillar proteins (MP) during storage, therefore preventing aggregation and denaturation (Wang et al., 2012). Meanwhile, their hydrophilic nature suggests the ability to inhibit ice crystal growth and restrict water mobility within protein networks via hydrogen bonding, ionic interactions and van der Waals forces. Uronic acid and other monosaccharides in the TFO structure permit scavenging of free radicals and prevent oxidation in protein molecules, which is a major reason for the quality deterioration of surimi during frozen storage (Hu et al., 2021). Despite these benefits, the efficacy of TFO as a cryoprotectant in surimi or seafood under frozen or fluctuating conditions remains unexplored. The possible synergistic mixture of EGCG and TFO could protect surimi and MP by creating a potential barrier around protein molecules. The TFO could provide water retention and bulk stabilization, while EGCG assists in antioxidative defense and protein stability at the molecular level.

In this study, the cryoprotective role of EGCG-TFO was investigated in snakehead fish surimi subjected to frozen storage cycles. We specifically assessed the impact of TFO on mitigating oxidative damage and preserving the structure of surimi proteins, which are key determinants of final product quality. Given the abundance of tea flowers and the critical need for effective natural cryoprotectants, the aim of this study was to stimulate interest in the surimi and seafood industries, potentially fostering cross-sector collaboration.

## 2 Materials and methods

### 2.1 Materials

Snakehead fish (number of fish 4, weight 2.5 ± 0.4 kg) were acquired from the fish market in Hangzhou, Zhejiang, China. Tea flowers were collected from the local garden of the College of Tea Science, Zhejiang A&F University. EGCG (food grade, CAS: 154–23-54, purity 98%) was purchased from Shaanxi Yuzhou Biotechnology Co., Ltd. Komio Reagent Co., Ltd. while China National Reagent Co., Ltd. supplied sucrose and sorbitol (food grade). The remaining chemicals were sourced from Sinophram Chemical Reagents Co., Ltd. located in Shanghai, China.

### 2.2 Extraction of tea flower oligosaccharides (TFO)

Dried tea flowers (*Camellia sinensis*) were ground into a fine powder. The powder was defatted by stirring with 80% ethanol (1:20, *w*/*v*) in a water bath at 70 °C for 1 h. After removing the supernatant, the residue was subjected to a second ethanol treatment. The resulting residue was collected by filtration and heated in an oven at 60 °C until completely dry. Hot water extraction was then performed on the defatted powder (1:20, w/v) for 2 h. This extraction was repeated, and the combined filtrates were concentrated under reduced pressure at 60 °C to approximately one-fifth of the original volume using a rotary evaporator. Absolute ethanol (5 times the volume of the concentrate) was added to precipitate the oligosaccharides, and the mixture was stored statically overnight at 4 °C. The precipitate was recovered by centrifugation (3000 rpm, 15 min), washed several times with absolute ethanol and dissolved in distilled water. Deproteinization was achieved using the method described by Chen et al. (2012), with minor alterations. Chloroform and n-butanol (4:1, *v*/v) were mixed with an aqueous oligosaccharide solution (1:5, v/v). The mixture was shaken vigorously for 30 min and centrifuged (3000 rpm, 10 min) to collect the supernatant. This deproteinization step was repeated 6–7 times until no protein interphase was visible. The final supernatant was filtered through a 700-mesh gauze. The filtrate was freeze-dried, and the resulting powder was ground to obtain purified TFO, which was stored in a desiccator until further use (the preparation of tea flower oligosaccharides is shown in the Supplementary data, Fig. S1).

### 2.3 Characterization of tea flower oligosaccharides (TFO)

A TFO solution (1 mg/mL) was prepared for compositional analysis. The total sugar and reducing sugar contents were 76.92 ± 0.05 and 13.73 ± 0.002%, respectively. Based on these values, the average degree of TFO fraction polymerization (DP) was calculated and to be 5.6.

### 2.4 Preparation of complexes (EGCG:TFO = 2:1)

EGCG (10 g) and TFO (5 g) were dissolved in 200 mL of ultrapure water, stirred magnetically for 30 min and then freeze-dried. After grinding, the obtained EGCG-TFO complexes were stored in a desiccator until experimental use. EGCG:TFO (2:1) was selected based on preliminary analyses, such as reducing capacity, DPPH and ABTS (as shown in the ‘Supplementary data’: Tables S1 and S2, Figs. S2 and S3).

### 2.5 Surimi and myofibrillar protein preparation

Surimi and MP were prepared according to the method described by Walayat, Wei, Su, Li, et al. (2024); Walayat, Wei, Su, Lorenzo, & Nawaz, 2024, with some modifications. Deceased snakehead fish (*Channa striata*) were processed by removing their skin and bones. The dorsal muscles were excised, cut into small pieces and rinsed 2–3 times with ultrapure water containing 0.6% NaCl. After draining excess surface moisture, the muscle pieces were minced to produce surimi. Surimi was homogenized with a 5-fold volume (*v*/*w*) of ice-cold low-salt buffer (0.05 mol/L NaCl, 3.38 mmol/L NaH₂PO₄·2H₂O, 15.5 mmol/L Na₂HPO₄·12H₂O, pH ∼7.0), using a high-speed homogenizer (HUXI-HR-25D, Beijing, China). The homogenate was centrifuged (Thermo-Fisher, Sorvall ST 8R, Am Kalkberg, Germany) (3500 rpm, 10 min, 4 °C) and the pellet was collected. The pellet was washed twice by resuspension in low-salt buffer and centrifugation. The washed pellet was then homogenized in a 4-fold volume (v/w) of ice-cold high-salt buffer (0.45 mol/L NaCl, 3.38 mmol/L NaH₂PO₄·2H₂O, 15.5 mmol/L Na₂HPO₄·12H₂O, pH ∼7.0) and stored at 4 °C for 12 h to ensure complete protein solubilization. After centrifugation (3500 rpm, 10 min, 4 °C), the supernatant was collected. Myofibrillar proteins (MP) were precipitated by diluting the supernatant with 10 volumes (*v*/v) of ice-cold distilled water and then centrifuging once more. Finally, the MP pellets were collected. The protein concentration of the MP suspension was 128.87 mg/mL, as determined via the biuret method proposed by Gornall et al. (1949).

### 2.6 Sample preparation and treatment

Aliquots of the MP suspension were prepared base on protein concentrations: Experimental Groups: supplemented with 1% and 3% EGCG-TFO complex; Positive Control Group (K): supplemented with 4% sorbitol +4% sucrose, which is considered as an industrial standard; Blank Control Group (C): no additives were administered. Similarly, four groups of surimi samples were prepared based on the above treatments (C, K and EGCG-TFO (1 and 3%)). These samples underwent 0, 2, 4, 6 and 8 FCY. One FCY comprised of samples stored at −18 °C for 3 days, which were then transferred to 4 °C for 24 h.

*Note:* The MP samples were also incorporated with EGCG (1 and 3%) and TFO (1 and 3%) and analyzed along C, K, EGCG-TFO (1% and 3%) for total sulfhydryl contents, Ca^2+^ATPase activity, carbonyl contents and surface hydrophobicity to provide a comprehensive comparison with EGCG-TFO (1 and 3%) combination. (as shown in supplementary data: S4 to S7)

### 2.7 Total sulfhydryl content

Total sulfhydryl content was measured according to the method by Sun et al. (2023), with certain modifications. The MP samples were mixed with 0.1 M of a phosphate buffer (pH 6.8). An aliquot (1 mL) of this mixture was combined with a solution containing 0.6 M KCl, 8 M urea, 2% SDS and 10 mM EDTA (pH 6.8). After centrifugation (10,000 ×*g* for 10 min), 0.5 mL of Ellman's reagent was added to the supernatant. The absorbance was measured at 412 nm using a TU1900 UV-spectrophotometer (Beijing, China). All samples were prepared in triplicate and TSH content was calculated using a molar extinction coefficient of 13,600 M^−1^ cm^−1^, which was expressed as mmol/kg protein.

### 2.8 Ca^2+^ATPase activity

Ca^2+^ATPase activity was determined using a modified version of the method proposed by Wang et al. (2023). A reaction mixture (2 mL) containing MP samples (CG, PCG, 1% EGCG-TFO and 3% EGCG-TFO), at a concentration of 3 mg/mL, was incubated. The reaction was stopped by the addition of trichloroacetic acid (100 g/L) to the reaction mixture. After centrifugation (2500 ×*g*, 5 min, 4 °C), inorganic phosphate release was measured. Ammonium molybdate (3 mL, 0.75 mol/L in H₂SO₄) and FeSO₄ (0.5 mL, 100 g/L in 0.15 mol/L H₂SO₄) were added to the supernatant, and the absorbance was measured at 700 nm. The activity was expressed as μmol of inorganic phosphate released per mg of protein per min. The samples were prepared in triplicate and Ca^2+^ATPase activity was assessed.

### 2.9 Carbonyl content

Carbonyls were measured based on the method suggested by Walayat, Wei, Su, Li, et al. (2024); Walayat, Wei, Su, Lorenzo, & Nawaz, 2024, with modifications. The MP samples were then diluted to a concentration of 2 mg/mL. The diluted sample (3 mL) was mixed with 3 mL of 2,4-dinitrophenylhydrazine (DNPH, in 2 mol/L HCl) and trichloroacetic acid. After centrifugation (3000 ×*g*, 8 min), the pellet was washed with 20% trichloroacetic acid and centrifuged again (2000 ×*g*, 10 min). The pellet was then washed with ethyl acetate:ethanol (1:1, *v*/v) and dissolved in 2 mL of 6 mol/L guanidine hydrochloride (in 20 mmol/L Na₃PO₄, pH 6.5). The absorbance was measured at 365 nm. CC was calculated using a molar extinction coefficient of 22,000 M^−1^ cm^−1^, which was expressed as nmol of carbonyl groups per mg of protein. All the biochemical assays were performed in triplicate.

### 2.10 Surface hydrophobicity

Surface hydrophobicity was assessed following the methodology given by Du et al. (2024), with minor adjustments. The MP samples were diluted to 3 mg/mL in 20 mM phosphate buffer (pH 6.0). The sample solution (1 mL) was mixed with bromophenol blue (BPB, 10 mg/mL) and shaken for 10 min. The mixture was then centrifuged (2000 ×*g* for 10 min). The absorbance of the supernatant was measured at 595 nm. Assessment of surface hydrophobicity for all samples was performed in triplicate, expressed as μg bound BPB, and calculated using the formula given in Eq. (1):

(1) $$\mathrm{BPB}\;\text{bound}\;\left(\mathrm{\mu g}\right)=200\times \left(A₅₉₅\text{control}-A₅₉₅\text{sample}\right)/A₅₉₅\text{control}$$

### 2.11 Fourier transform infrared (FTIR) spectroscopy

FTIR was performed following the method described by Sharma, Majumdar, Mehta, Ngasotter, and Gaurav (2024); Sharma, Majumdar, Mehta, Panda, & Ngasotter, 2024. The lyophilized MP powder was ground using liquid nitrogen and stored in a desiccator. FTIR spectra were acquired using an FTIR spectrometer (Thermo-Fisher, Nicolet iS50, Waltham, USA) over the range of 4000–400 cm^−1^. Each spectrum represented an average of 64 scans at a resolution of 4 cm^−1^, collected in the transmittance mode. Background scans were recorded before sample analysis. All samples were analyzed in triplicate.

### 2.12 Circular dichroism

The secondary structural attributes of the MP samples were determined following the protocol suggested by Walayat, Wei, Su, Li, et al. (2024); Walayat, Wei, Su, Lorenzo, & Nawaz, 2024. MP was diluted to a concentration of 0.05 mg/mL using a 0.6 M KCl buffer solution. The circular dichroism (J-1500-150, JASCO Co., 192–8537, Tokyo, Japan) parameters were set as follows: resolution (1 nm), wavelength (200–250 nm), sensitivity (50 millidegrees), response time (2 s), and scanning speed (10 nm), respectively.The MP samples were run in triplicate against 0.6 KCl blanks. The secondary structural attributes are presented in [θ] deg. cm^2^ dmol^−1^.

### 2.13 Scanning electron microscopy (SEM)

The microstructures of the surimi samples were observed using the method proposed by Sun et al. (2023), with some modifications. The surimi samples were cut into regular shapes (5 × 5 × 5 mm). The prepared samples were rinsed with a phosphate buffer (0.2 mol/L) and dehydrated with an ethanol series (50, 60, 70, 80, 90 and 100%) for 10 min three times. The dehydrated samples were freeze-dried, mounted and sputter coated with gold. The microstructure was analyzed using SEM (HITACHI, TM3030, Tokyo, Japan) with an acceleration voltage of 15 kV.All samples were prepared and analyzed in triplicate.

### 2.14 Molecular interaction of myosin with EGCG-GA (TFO)

To investigate the molecular dynamics of myosin with two different ligand molecules, molecular dynamic modeling was utilized.

#### 2.14.1 Alignment of sequences and template

The heavy chain sequence for myosin of snakehead fish has already been established (refer to entry in UnitProtKB). A myosin template; however, there is no structure available in the Protein Data Bank of myosin for this species from external sources (refer to the RCSB website). Consequently, the myosin heavy chain sequence has been reported as ‘myosin’ because of the lower likelihood of myosin having a probabilistically generated molecular dynamic model and successful comparison with others in the past. A suitable template for comparison in the current study was obtained from the Protein Data Bank (PDB ID: 1C1G), which contains highly similar myosin heavy chain structures. In this study, two ligand molecules were examined: epigallocatechin gallate (EGCG; CID 65064) and galactose (GA; CID 6036). The structures of these compounds were downloaded from the PubChem database and then optimized by ChemDraw 3D Version 19.1 (Perkin Elmer) using the MM2 function conditions.

#### 2.14.2 Preparing with homology modeling

A suitable reference protein (PDB ID: 1C1G) containing a myosin heavy chain similar to that of the snakehead was obtained from the Protein Data Bank. By aligning the amino acid sequences, it was determined that the corresponding amino acids between 1C1G and the snakehead shared 54% identity. Thus, 1C1G was a suitable reference protein to generate a computer aid for the construction of myosin. The results indicated that 1C1G shares 42% similarity with shrimp myosin and that this percentage is a solid basis for deriving the structure of myosin. Therefore, the software program, Accelrys software (San Diego, California), was subsequently employed to generate a 3D model of snakehead myosin using the reference structural template provided (PDB ID: 1C1G).

#### 2.14.3 Docking of ligand molecules

Molecular Operating Environment (MOE) docking software allowed for the addition of hydrogen atoms to the heavy chain structure of myosin and the removal of water to perform docking analyses. The D-TEP site finder tool of MOE was subsequently employed to find the binding pocket of the myosin heavy chain to study the potential binding of TFO based fraction galactose (GA) and EGCG. Once identified, the five best performing docking poses (for the analyzed ligands) were determined using the London Free Energy scoring method. In order to evaluate the forcefield-based approach, careful consideration was given to the rigid frame of the receptor (myosin) so that values could be calculated in Å. Root mean square deviation (RMSD) analyses were also performed on the best performing ligands to determine, which one of them best bound to the receptor's binding pockets. To investigate possible molecular interactions between ligand molecules and the receptor, the Lig-X tool of MOE was used to provide a visual representation of ligand/receptor interactions and locations. In addition to producing a visual representation of these ligand-receptor interactions, the software program gave rendered the 2D location of the stabilizing force between ligands and receptor binding pockets. The receptor ligands and snakehead were specified as receptors in the chemical docking models prior to docking myosin with the ligands.

#### 2.14.4 Molecular dynamic simulations (MDs)

Utilizing GROMACS software version 5.0, Molecular Dynamic Simulations (MDs) were performed on myosin chains dissolved in either water or myosin-EGCG-GA solution. As one of the initial processes, each MD system was minimized by the steepest descent method(s). Each MD simulation was run using a time-step of two (2) fs for a total of one (1) ns for equilibration temperature/volume/number of equivalent particles. At the end of the initial temperature equilibration, each MD system was continuously maintained at a temperature of three hundred ten (310) K for another one (1) minute of equilibrium. The pressure of each MD system was maintained at one (1) atm using a Parrinello-Rahman Barostat with a two (2) ps coupling time during equilibration. Each MD system used periodic boundary conditions in all three (3) dimensions with semi-isotropic pressure coupling for the x- and y-dimensions and dissimilar z-dimension coupling. To create a solvent environment for each MD system, the center of each cubic box was at least twelve (12) nm from the nearest protein atom, with the cubic boxes containing TIP3P water. Root mean square deviations (RMSD) of all systems demonstrated symmetry of their trajectories within 0.5 Å during the last 100 ns of simulation time. The last one hundred (100) ns of each trajectory was used to determine the positions and orientations of myosin chains. Ten (10) independent MD simulation runs of one hundred (100) ns were undertaken for each MD system, with each MD run done in duplicate four (4) times. Truncated distances of 1.2 nm were employed to simulate the electrostatic potential energy as calculated using the Particle-Mesh Ewald technique.

### 2.15 Statistical analysis

Statistical analysis was performed using SPSS, Version 21.0. The results were calculated based on triplicate values, and the mean ± SD was extracted. Prior to analysis, data were tested for normal distribution and homogeneity of variance to meet the assumptions of ANOVA. The values were subjected to Duncan's method (multiple comparison test) along with one-way ANOVA at a 0.05 significance level and a confidence interval time of 95%. Specific *P*-values and effect sizes are provided in the supplementary data (Table S3). All graphs were prepared using OriginPro, version 2025.

## 3 Results and discussion

### 3.1 Total sulfhydryl contents

**Fig. 1:**
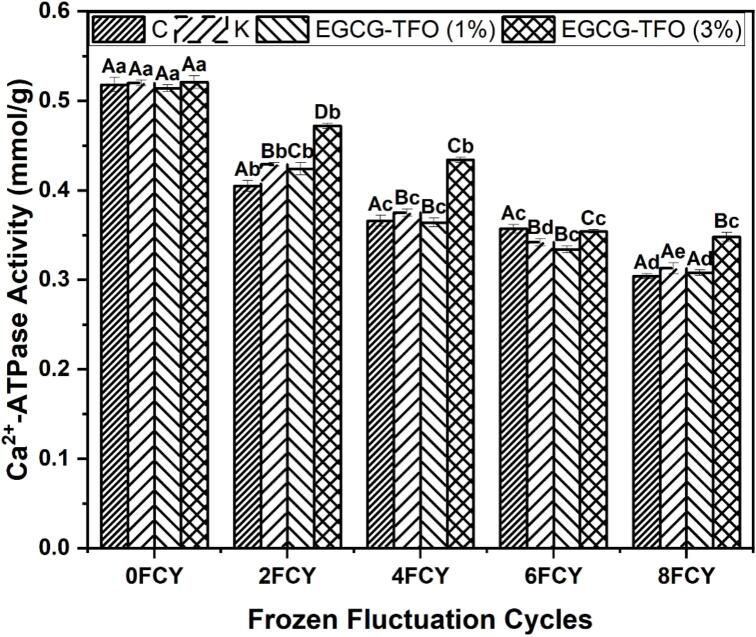
Ca^2+^ATPase activity of MP treated with C, K, EGCG-TFO (1%) and EGCG-TFO (3%) during frozen fluctuation cycles. Upper case letters (A–D) on error bars show significant differences (*P < 0.05*) in different treatment within the same freeze-thaw cycle. Lower case letters (a–e) on error bars show the individual treatment within the different freeze-thaw cycles.

**Fig. 2:**
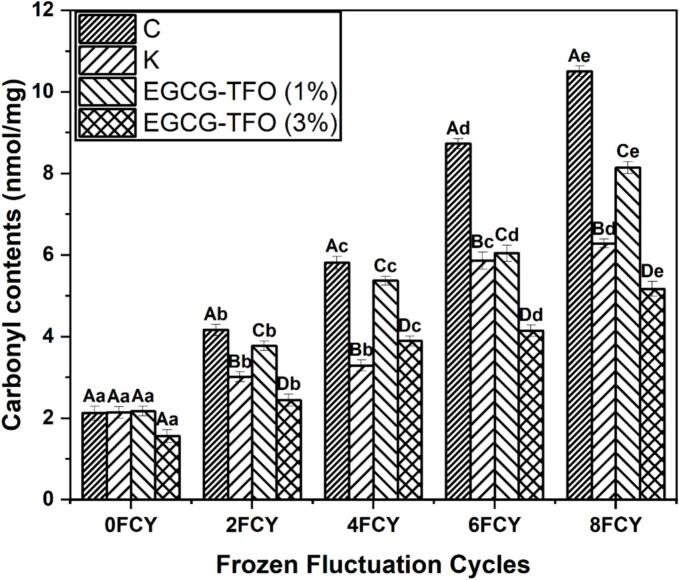
Carbonyl contents of MP treated with C, K, EGCG-TFO (1%) and EGCG-TFO (3%) during frozen fluctuation cycles. Upper case letters (A–D) on error bars show significant differences (*P < 0.05*) in different treatment within the same freeze-thaw cycle. Lower case letters (a–e) show the individual treatment within the different freeze-thaw cycles.

The total sulfhydryl content is key in determining the exposure of protein functional sites to an oxidative environment. The overall changes in the total sulfhydryl content in all MP samples (C, K, and EGCG-TFO (1 and 3%)) are shown in Table 1. Despite the addition of MP with K and EGCG-TFO (1 and 3%), there was a notable decrease in the total sulfhydryl content during 8 FCY. A dominant decline in total sulfhydryl content was observed in the C samples with no cryoprotectants, from 54.68 to 34.70 (mmol/kg) after 8 FCY (Table 1). The normal distribution and homogeneity of variance are added in the supplementary data (Table S3). In addition, the K-added MP also demonstrated a drop in total sulfhydryl content from 54.93 to 37.16 (mmol/kg), but it was more stable than in the C samples. This notable decrease in the total sulfhydryl content may be associated with the unfolding of protein molecules and the conversion of sulfhydryl to disulfides (Wang et al., 2022). This possible mechanism of myosin denaturation may have occurred because myosin is exposed to an oxidative environment, leading to structural disintegration and conformational instability (Sun et al., 2023). Moreover, the loss of total sulfhydryl content can also be related to disulfide formation among the cysteine regions of myosin (Wang et al., 2022). Another possible reason for the decline in total sulfhydryl content may be associated with fluctuations in pH and lipid oxidation, which further accelerate chemical modifications (intermolecular interactions) and oxidation (Sun et al., 2024). In contrast, the (1%) mixed MP also showed a drop in total sulfhydryl content from 54.49 to 35.29 (mmol/kg), which is even lower than that noted for the K added samples. Meanwhile, the EGCG-TFO (3%)-added MP exhibited a more stable decline in total sulfhydryl content from 54.68 to 39.50 (mmol/kg) after 8 FCY, which is better stability than in the case of the C, K and EGCG-TFO (1%) treatments. The current findings show that the inclusion of EGCG-TFO (3%) into the protein matrix (MP) protects cysteine by creating hydrogen bonds, forming electrostatic interactions, and through antioxidative radical scavenging (Wang et al., 2022). In parallel, samples supplemented with EGCG (1% and 3%) and TFO (1% and 3%) were studied. The EGCG-TFO (3%) combination showed a possible reduction in the decline in total sulfhydryl content over all other samples; however, EGCG (3%) was found to protect against oxidative damage more effectively than both EGCG (1%) and TFO (1% and 3%) (refer to supplementary data and Table S4). Prior studies suggest that the incorporation of oligosaccharides can limit the decrease in total sulfhydryl level through the mechanisms of scavenging, antioxidant and dynamic hydrogen binding between oligosaccharides and amino acids (Mo et al., 2024). As reported by Zheng et al. (2023), the incorporation of *velutipe* polysaccharides prevented the total sulfhydryl content in catfish myofibrillar proteins due to their well-established hydrophobic interactions and minimization of the amount of unbound water molecules in the protein matrix. Walayat, Wei, Su, Li, et al. (2024); Walayat, Wei, Su, Lorenzo, & Nawaz, 2024 also found that a tea polysaccharide concentration of 3% is effective in preserving the total sulfhydryl content in silver carp during frozen temperature fluctuations compared to the control and positive control samples by inhibiting the conversion of total sulfhydryl content into disulfide groups. According to Tian et al. (2022), oligosaccharides function primarily via molecular scale interactions between amino acid residues and oligosaccharides, especially those sites containing cysteine, which prevents a decrease in sulfhydryl levels. These findings are in agreement with the following: Ca^2+^ATPase (Fig. 1) and carbonyl content (Fig. 2), where EGCG-TFO (3%) restricted oxidative and conformational changes in MP during FCY.

**Table 1:** Total sulfhydryl contents of MP treated with C, PK, EGCG-TFP (1%) and EGCG-TFP (3%) during frozen fluctuation cycles.

Treatments/FCY	C	K	EGCG-TFP (1%)	EGCG-TFP (3%)
0 FCY	54.68 ± 0.55^Aa^	54.93 ± 0.31^Aa^	54.49 ± 0.08^Aa^	54.68 ± 0.78^Aa^
2 FCY	52.04 ± 0.46^Ab^	53.55 ± 0.52^Bb^	53.47 ± 0.68^Bb^	52.4 ± 0.68^ABb^
4 FCY	48.59 ± 0.21^Ac^	42.41 ± 0.16^Bc^	42.87 ± 0.24^Bc^	45.86 ± 0.49^Cc^
6 FCY	44.64 ± 0.26^Ad^	39.90 ± 0.65^Bd^	38.46 ± 0.56^Cd^	43.70 ± 0.10^Dd^
8 FCY	34.70 ± 0.43^Ae^	37.16 ± 0.11^Be^	35.29 ± 0.85^Ce^	39.50 ± 0.22^De^

Error bars show the standard deviation (SD) of three replicate measurements Upper case letters (A-D) show significant differences (*P < 0.05*) in different treatments within the same freeze-thaw cycle. Lower case letters (a-e) show the individual treatment within the different freeze-thaw cycles.

### 3.2 Ca^2+^ATPase activity

Ca^2+^ATPase activity is a key parameter used to investigate FCY-induced oxidation and related MP changes. As shown in Fig. 1, despite treatment with EGCG-TFO and the conventional mixture (K), all MP samples showed a major decline in Ca^2+^ATPase activity from 0 to 8 FCY.Among all the MP samples, C samples without any addition of cryoprotectant showed the highest decline in Ca^2+^ATPase activity, from 0.518 to 0.304 (mmol/g). The normal distribution and homogeneity of variance are added in the supplementary data (Table S3). A similar declining trend was also observed in the K treated MP from 0.521 to 0.313 (mmol/g), but this drop in Ca^2+^ATPase activity was more stable than in the samples without any treatment. This decrease in Ca^2+^ATPase activity indicates possible changes in the myosin head and tail regions due to FCY. This decline can be linked to the formation of ice crystals caused by the availability of unbound water in the protein matrix, leading to larger ice crystals and protein denaturation in FCY (Sun et al., 2024). Meanwhile, Chen et al. (2022) also suggested that this Ca^2+^ATPase activity decline may be due to significant exposure of myosin amino residues, such as tryptophan and tyrosine, due to changes in the frozen storage temperature accelerating oxidation. These oxidative changes lead to the breakdown of hydrogen and ionic interactions between protein molecules, resulting in protein aggregation and an increased amount of water molecules in the protein system. Notably, the EGCG-TFO (1 and 3%)-treated MP also demonstrated a decrease in Ca^2+^ATPase activity during 8 FCY (Fig. 1). The decline in Ca^2+^ ATPase activity in EGCG-TFO (1%)-treated MP was greater than that in K MPs, which may be associated with the lower concentration of EGCG-TFO, but might not be sufficient to effectively stabilize FCY-induced oxidation. The decline observed for EGCG-TFO (1%) was from 0.514 to 0.308 mmol/g, and for EGCG-TFO (3%), it was from 0.521 to 0.348 mmol/g, respectively (Fig. 1). These outcomes suggest that the treatment of MP with EGCG-TFO (3%) can be effective in maintaining overall integrity against oxidative changes, such as the restricted deterioration of Ca^2+^ATPase activity during FCY. Furthermore, samples that were treated with EGCG (1% and 3%) and TFO (1% and 3%) were analyzed, where the EGCG-TFO combination (3%) demonstrated increased stability in Ca^2+^ATPase activity compared to all other samples, whereas the Ca^2+^ATPase activity associated with EGCG (3%) decreased at a lower rate than EGCG (1%) and TFO (1% and 3%) (shown in supplementary data: Table S5). The possible protective mechanism of EGCG-TFO (3%) might be due to potential antioxidative, shielding attributes and intermolecular interactions (e.g., hydrogen and ionic interactions) with amino acids, thereby preventing water mobility and oxidative alterations (Sun et al., 2024). These protective properties may enhance Ca^2+^ATPase activity owing to potential radical scavenging, molecular stability and EGCG-TFO binding with myosin conformational sites (Chen et al., 2022). This restricted physical disruption of the protein matrix may be linked to the formation of an EGCG-TFO protective shield around the protein molecules and its effectiveness against nucleation and water mobility. Xie et al. (2025) investigated the effect of tamarind seed polysaccharides on silver carp proteins, where the addition of 3% tea polysaccharides restricted the decline in oxidative induced changes during frozen storage temperature fluctuations. The addition of polysaccharides as cryoprotectants also prevents Ca^2+^ATPase activity by restricting oxidative changes in the myosin region.

### 3.3 Carbonyl contents

The changes in carbonyl content in all MP samples (C, K, EGCG-TFO (1 and 3%)) during 8 FCY are shown in Fig. 2. As noted, a substantial increase in carbonyl content was observed in all MP samples (C, K and EGCG-TFO (1 and 3%)). The major increase in carbonyl can be seen in the C samples, ranging from 2.12 to 10.50 (nmol/mg) after 8 FCY. The normal distribution and homogeneity of variance is added in the supplementary data (Table S3). This possible increase in carbonyls may be due to accelerated oxidation within protein molecules during FCY, leading to weaker interactions among functional amino acids and aggregation of protein molecules (Wang et al., 2022). Moreover, these alterations lead to further deteriorative changes in NH and NH_2_ binding, resulting in an increased carbonyl content (Xu & Xu, 2021). Meanwhile, a significant upsurge in carbonyls was noted in the K group from 2.14 to 6.28 (nmol/mg), indicating FCY-induced oxidative changes in proteins despite the addition of a conventional cryoprotectant combination. Interestingly, the increase in carbonyls of EGCG-TFO (1%)-treated MP was more dominant than that of K-treated MP, indicating that the correct concentration of EGCG-TFO was necessary to maintain the carbonyl content during 8 FCY. Furthermore, the addition of EGCG-TFO (3%) to MP showed more promising stability of carbonyls, from 1.56 to 5.17 (nmol/mg) after 8 FCY, which was better than that of the C, K and EGCG-TFO (3%) treated MP samples (Fig. 2). This possible inhibition of carbonyl increase is linked to the potential shielding effect of EGCG-TFO against oxidative induced changes and suppressed reactive oxygen species (Wang et al., 2022). In addition, the stronger interactions of EGCG-TFO (3%) could decrease the availability of free water molecules in the protein system, establishing electrostatic and hydrogen bonds, as well as reducing the oxidative environment (Zhang et al., 2023). Furthermore, this research included the findings of EGCG (1 and 3%) and TFO (1 and 3%) along with C, K and EGCG-TFO (1 and 3%); results are provided in supplementary data: Table S6. Ling et al. (2023) reported that the addition of polysaccharides proteins can prevent the increase in oxidatively produced carbonyls due to their effective hydrophilic nature, minimizing the unbound water molecules, which leads to reduced oxidative alterations and ultimately inhibits the increase in carbonyls. In earlier studies, it has also been stated that the addition of oligosaccharides is effective in stabilizing protein quality and inhibiting carbonyls during freezing temperature changes (Xu & Xu, 2021). These results indicate that the addition of EGCG-TFO (3%) may extend snakehead quality during FCY by preventing the oxidation of amino acid side chains (e.g., lysine, proline, thionine and arginine). These findings coincided with a decline in Ca^2+^ATPase activity, indicating myosin denaturation (Fig. 1).

### 3.4 Surface hydrophobicity

**Fig. 3:**
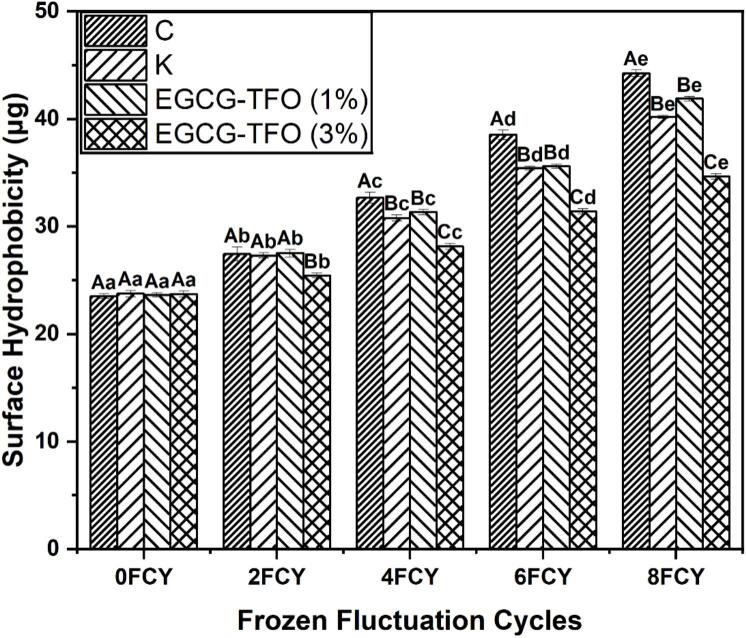
Surface hydrophobicity of MP treated with C, K, EGCG-TFO (1%) and EGCG-TFO (3%) during frozen fluctuation cycles. Upper case letters (A–D) on error bars show significant differences (*P < 0.05*) in different treatment within the same freeze-thaw cycle. Lower case letters (a–e) the individual treatment within the different freeze-thaw cycles.

Surface hydrophobicity (S_0_) is an important indicator of conformational and structural alterations in the protein matrix during FCY. The increase in S_0_ was assessed in all groups (C, K EGCG-TFO (1 and 3%)) during the 8 FCY (Fig. 3). The highest increase in S_0_ among all MP samples was observed in the C samples with no cryoprotectants (from 23.52 to 44.25 μg after 8 FCY). The normal distribution and homogeneity of variance is added in the supplementary data (Table S3). Meanwhile, for the K-added MP samples, a substantial upsurge was also found, but it was more restricted compared to the C samples (from 23.75 to 40.19 μg after 8 FCY), indicating low cryoprotective abilities of the conventional combination of sucrose and sorbitol. This increase in S_0_ during 8 FCY reflects oxidation-induced amino acid unfolding and conformational alterations (Ling et al., 2023). This may be due to the exposure of the hydrophobic interior to an oxidative environment, leading to weaker interactions between the amino acids and Amide *I* and II regions (Shen et al., 2022). Therefore, it can be concluded that FCY has a direct influence on the increase in S_0_ and is linked to the structural as well as interactive integrity of amino acids in the protein matrix. Furthermore, this increase in S_0_ could also be associated with the formation of hydrophobic linkages between functional amino acids, leading to the coagulation and disintegration of proteins. The sequence of these changes, such as amino acid exposure to oxidation, irregular ice crystals and coagulation, may increase S_0_ due to the burial of the hydrophobic region and revelation of the hydrophilic region within the protein aggregates (Wu et al., 2024). On the other hand, MP added with EGCG-TFO (1 and 3%) also demonstrated a rise in S_0_ during 8 FCY, but this increase was more restricted than that of the C MP samples. Notably, the EGCG-TFO (1%) showed a significant increase compared to the K treated MP, from 23.62 to 41.87 μg, which is consistent with our earlier findings for Ca^2+^ATPase (Fig. 1) and carbonyl contents (Fig. 2). The increased S_0_ in the EGCG-TFO (1%) samples compared to the K-added MP could have been caused by the lower concentration of EGCG-TFO, which is apparently ineffective in minimizing the increase in S_0_. Meanwhile, the EGCG-TFO (3%) mixed MP exhibited better stability in S_0_ than the C, K or EGCG-TFO (1%) MP samples, with an increase from 23.71 to 34.65 μg. This maintained S_0_ in EGCG-TFO (3%) samples associated with hindered oxidation and water redistribution within the protein matrix due to potential cross-linking between myosin and EGCG-TFO (Wu et al., 2024). In addition, EGCG-TFO (3%) appears to be an effective concentration for mitigating protein coagulation and oxidation (Shen et al., 2022).The improved stability in S_0_ by EGCG-TFO (3%) is due to molecular scale interactions between protein functional groups and EGCG-TFO and antioxidative mediated suppression of oxidative unfolding of protein molecules (Wu et al., 2024). The analysis of the surface hydrophobicity of MP samples added with EGCG (1 and 3%) and TFO (1 and 3%) along with C, K and EGCG-TFO (1 and 3%) is shown in supplementary data S7 for comprehensive comparison. Previous studies have been confirmed that the addition of tea polysaccharides can prevent the rise in S_0_ by inhibiting oxidation and forming hydrophilic-hydrophilic interactions (Walayat, Wei, Su, Li, et al., 2024; Walayat, Wei, Su, Lorenzo, & Nawaz, 2024). On the other hand, the stabilization noted with EGCG-TFO in this study is most likely due to its potential synergistic effect, greater ability to act as an antioxidant, and shielding effect (Ling et al., 2023). Walayat, Wei, Su, Li, et al. (2024); Walayat, Wei, Su, Lorenzo, & Nawaz, 2024 also noted that the addition of tea polysaccharides stopped the increase in S_0_ by improving ionic binding with the functional sites of protein molecules. In conclusion, EGCG-TFO (3%) was helpful in maintaining the integrity of aquatic proteins by stabilizing the increase in S_0_ during FCY.

### 3.5 Fourier transform infrared spectroscopy

**Fig. 4:**
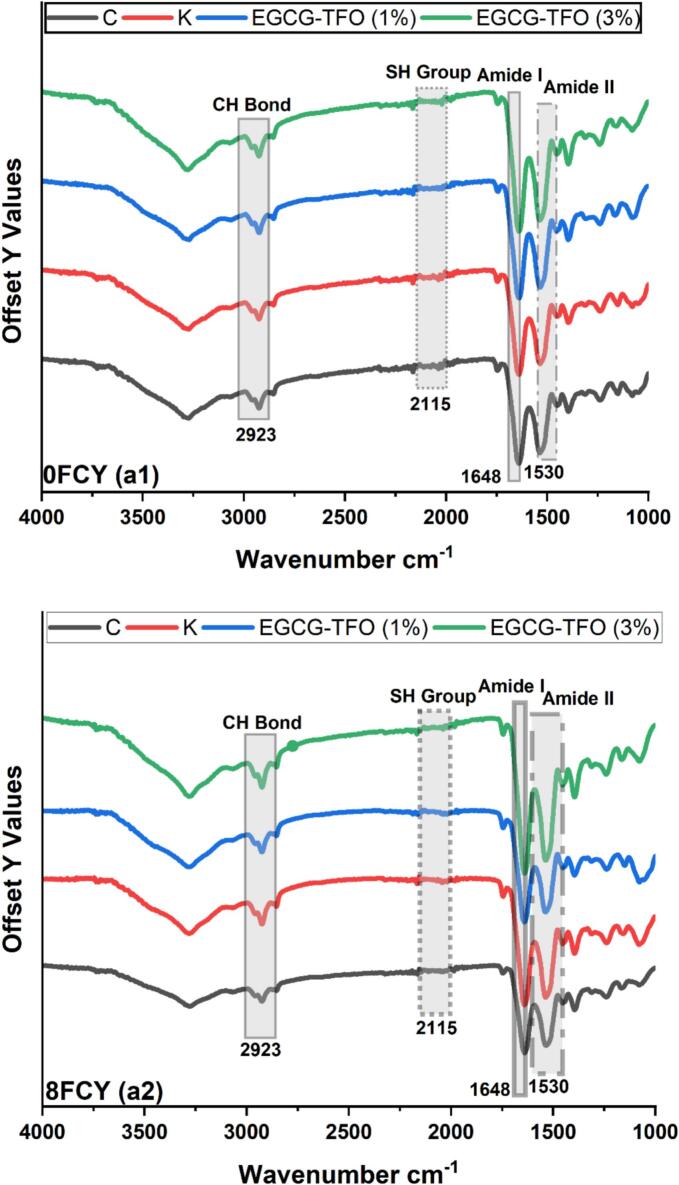
FTIR of MP treated with C, K, EGCG-TFO (1%) and EGCG-TFO (3%) during frozen fluctuation cycles. Upper case letters (A–D) on error bars show significant differences (*P < 0.05*) in different treatment within the same freeze thaw cycle. Lower case letters (a–e) show the individual treatment within the different freeze-thaw cycles; where: 2920 cm^−1^ represent CH bonds, 2100 and 2150 cm^−1^ SH content, 1650 and 1540 cm^−1^ Amide *I* and II, respectively.

Fourier transform infrared (FTIR) spectroscopy is a critical tool for assessing alterations in the structural components of MP during FCY. The structural component alterations of MP treated with C and K EGCG-TFO (1 and 3%) are shown in Fig. 4 (a1 and a2). All MP samples (C, K and EGCG-TFO (1 and 3%)) demonstrated a typical spectral trend, suggesting alterations in different structural attributes after 8 FCY. The three most relevant spectral wavelengths are specified in Fig. 4 (a1 and a2), with pointed intensities based on the linkage between oxidation and structural attributes. As mentioned in earlier studies, 2920 cm^−1^ is associated with CH bonds (Sharma, Majumdar, Mehta, Panda, & Ngasotter, 2024). During these 8 FCY, samples without any treatment presented a change in the intensity spectra at 2923 cm-^1^, indicating a variation in CH bonds. This alteration in the CH bond may occur because of the unfolding of proteins after the linkage of their interaction with methylene, which possesses more electronegative charges and is directed to the modification of the CH bond, and consequently, variation in the spectral intensity in the 2923 cm^−1^ regions (Walayat, Wei, Su, Li, et al., 2024; Walayat, Wei, Su, Lorenzo, & Nawaz, 2024). Similar changes in the CH bond intensity were also prominent in the MP samples treated with K and EGCG-TFO (1%). In contrast, EGCG-TFO (3%) showed stability in CH bonds against 8 FCY, which is aligned with our above findings (Fig. 1, Fig. 2, Fig. 3). In addition, another important intensity spectrum exists between 2100 and 2150 cm^−1^, which indicates the amount of SH content available in this specific region and is reported to be prone to an oxidative environment (Tian et al., 2022).In the current findings, a change in intensity was observed at the spectral region of 2115 cm^−1^ and thereafter an alteration in spectral intensity (as shown in Fig. 4 a2), which is indeed a minor alteration but pointing towards the reduction of SH groups, which confirms our above findings of total sulfhydryl contents (Table 1). This absolute modification of the SH groups is due to oxidative conversion into disulfides and S—S groups in the protein system (Mo et al., 2024). A similar trend of declining SH groups was observed in the other MP samples (K and EGCG-TFO (1%)), whereas for EGCG-TFO (3%), a consistent intensity wavelength was recorded at 2115 cm^−1^. Furthermore, another important spectral region is Amide *I* and II, which exist at 1540 and 1650 cm^−1^, respectively, denoting modifications in tyrosine, tryptophan, α-helix contents, and N—H and C—N bonding (Walayat, Wei, Su, Li, et al., 2024; Walayat, Wei, Su, Lorenzo, & Nawaz, 2024). As shown in Fig. 4, a visible change at 1530 cm^−1^ was noted in the MP samples (C, K and EGCG-TFO (1%)), suggesting a drop in N—H bonds, which is important for analyzing the initiation of the oxidation process (Nunez-Flores et al., 2018). Meanwhile, the EGCG-TFO (3%) MP samples displayed a minor variation at the spectral region of 1530 cm^−1^ compared to the C, K and EGCG-TFO (1%) MP samples. The spectral modifications in this region (1530 cm^−1^) describe the alterations in hydrogen bonds among functional amino acids, which may be connected with the Amide II region. Furthermore, another significant spectral region exists at 1530 cm^−1^, which is linked to the variation in the N—H bonds and oxidation of the cysteine region in myosin. Another important spectral change at 1650 cm^−1^, attributed to Amide *I*, indicates the alteration of α-helix and β-sheets and overall protein structural stability. The change at 1650 cm^−1^ (Amide *I*) indicates protein aggregation and unfolding of conformational protein molecules during FCY. Moreover, a major variation at the 1650 cm^−1^ spectral wavelength was noted in C, which is more dominant than other MP samples (K and EGCG-TFO (1 and 3%)), indicating a decline in α-helix content. Meanwhile, EGCG-TFO (3%) showed stability at 1650 cm^−1^ spectral intensity by establishing potential hydrogen and ionic interactions with protein molecules and the possibility of preserving the myosin region against oxidation (Sharma, Majumdar, Mehta, Ngasotter, & Gaurav, 2024; Sharma, Majumdar, Mehta, Panda, & Ngasotter, 2024).

### 3.6 Circular dichroism (secondary structure)

**Fig. 5:**
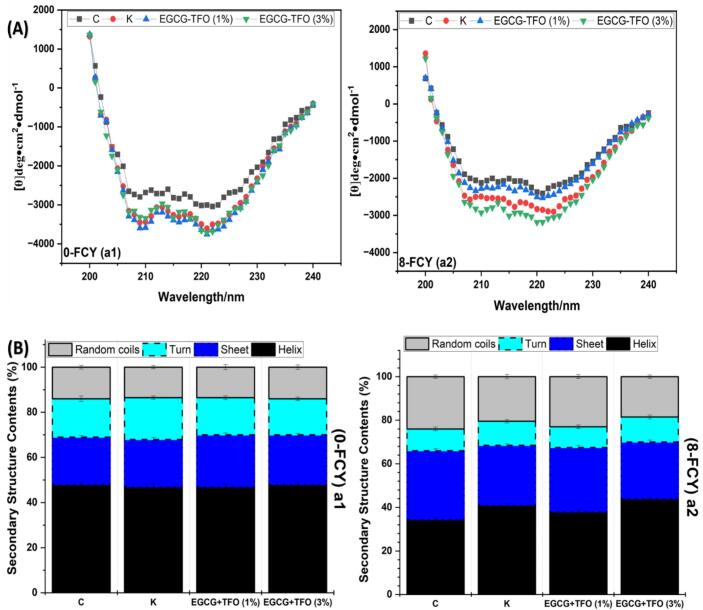
CD spectra of MP treated with C, K, EGCG-TFO (1%) and EGCG-TFO (3%) during frozen fluctuation cycles. (**A**) change in CD spectra from 200 to 240 nm (**B**) change in secondary structural properties.

Circular dichroism is a key technique for analyzing the secondary structural components, including α-helix, β-sheet, β-turn and random coil, as well as their related changes during frozen storage or temperature fluctuations. All samples (C, K and EGCG-TFO (1 and 3%)) are shown in Fig. 5A (a1 and a2) and B (a1 and a2). The circular dichroism revealed two important spectral regions at the band wavelengths of 208 and 222, used to assess alterations in α-helix content, which is crucial for MP structural integrity due to its presence in myosin, accounting for 95% of its total content (Wang et al., 2023). From 0 to 8 FCY, all samples C, K and EGCG-TFO (1 and 3%) showed a visual decline in the circular dichroism spectra at 208 and 222, despite treatment with the industrial or EGCG-TFO mixtures. The C samples exhibited a significant reduction in α-helix (48 to 34.5%) and an increase in β-sheets (21 to 32%), respectively (as shown in Fig. 5A (a1 and a2)). This decline in α-helix indicates disruption of the myosin region and hydrogen bonds, leading to an increase in β-sheets and unfolded protein molecules (Sun et al., 2022). In addition, Shang et al. (2022) stated that an increase in β-sheets and random coils might result from conformational changes in amino acids and protein aggregate formation. The formation of disulfides and exposure of hydrophobic residues may promote β-sheet content and contribute to protein aggregation and denaturation (Wang et al., 2023). Meanwhile, the K samples exhibited a prominent drop in α-helix from 47 to 41%, which was a notably smaller decrease compared to the EGCG-TFO (1%) samples, which decreased from 47 to 38%. Additionally, an increase in β-sheets was noted in both treatments (as shown in Figs. 5A (a1 and a2) and 5B (a1 and a2)). This stability in α-helix content after the addition of K and EGCG-TFO (1%) highlights the importance of cryoprotectants in maintaining the secondary structure integrity of MP. Furthermore, EGCG-TFO (3%) demonstrated significant stability in α-helix content (from 48 to 44%), and a smaller increase in β-sheets (22 to 26%) compared to the C, K and EGCG-TFO (1%) treatments. This improved structural stability aligns in EGCG-TFO (3%) refers to the molecular interaction between EGCG-TFO (3%) with myosin amino acid residues and mitigated oxidation (Tian et al., 2022). In earlier studies, it has also been shown that the addition of oligosaccharides and polysaccharides to MP can reduce the decline in secondary structural integrity by preventing oxidative alterations in the myosin region during frozen storage (Sun et al., 2024). These circular dichroism findings closely align with the FTIR results (Figs. 4 a1 and a2).

### 3.7 Scanning electron microscopy

**Fig. 6:**
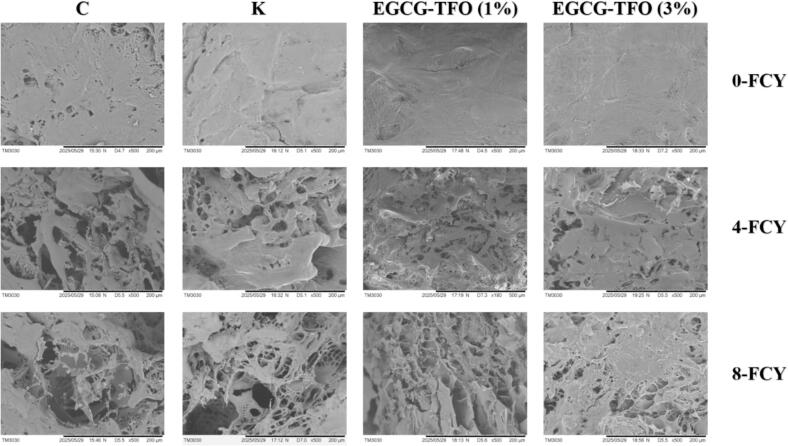
SEM of surimi treated with C, K, EGCG-TFO (1%) and EGCG-TFO (3%) during frozen fluctuation cycles; where: magnifying information .

Microstructural analysis was performed to analyze protein denaturation and aggregation at the micro-level during 8 FCY. The changes in the microstructure of surimi treated with C, K and EGCG-TFO (1 and 3%) are shown in Fig. 6 (a1, a2 and a3) after 8 FCY. All surimi samples demonstrated visible variability in microstructure from 0 to 8 FCY. The untreated C samples presented major deformation of the microstructure due to FCY-induced oxidation, denaturation and aggregation of protein molecules. Meanwhile, C samples showed larger cracks, porosity and protein fragments, which could be due to the disintegration of myosin and the formation of irregular and larger ice crystals during FCY (Ling et al., 2023). These structural damages reflect the unfolding of protein chains and molecular level oxidative disruption. Moreover, the K surimi samples supplemented with the conventional cryoprotective mixture exhibited similar microstructure disorientation; however, these microstructural alterations were fewer than those in the C samples. This K treatment showed stability in the microstructure during FCY, but was not efficient in firming the microstructure after multiple FCY. Importantly, the EGCG-TFO (1%) treated samples showed major microstructural alterations (denaturation, aggregation and disintegration of protein interactions) compared to the K samples. This weaker protection of EGCG-TFO (1%) could be due to the application of EGCG-TFO at a lower concentration in surimi, which is not sufficient for the preservation of snakehead fish microstructural attributes. Furthermore, surimi containing EGCG-TFO (3%) exhibited significantly more stable microstructural properties (as shown in Figures 6a1, a2 and a3) than C, K and EGCG-TFO (1%) samples. Moreover, EGCG-TFO (3%) assisted in preserving the overall structural integrity, interlinking the protein molecules and inhibiting the amount of water in the protein matrix. The well-connected and dense microstructure of the EGCG-TFO (3%)-treated samples could be due to its antioxidative effect against oxidative radicals, which prevents the coagulation of proteins and intermolecular binding interactions (Zhu et al., 2022). In addition, the enhanced quality of the molecular structure is the result of the enhanced interaction between the molecular arrangement of EGCG-TFO and the amino acid sites on the protein, which is accomplished through hydrogen bonding and electrostatic interactions, and through the general protective nature of EGCG-TFO as an antioxidant for preventing the aggregation of protein molecules by oxidative radicals. Furthermore, the ability of EGCG-TFO restricts the movement of water in the protein matrix, thereby decreasing environmental stress within the protein matrix and maintaining closely spaced and suitably arranged protein structures. In previous studies, it has also been reported that the addition of tea polysaccharides can extend the structural stability of aquatic proteins during frozen temperature fluctuations (Ling et al., 2023). Xie et al. (2025) noted that the addition of tamarind polysaccharide to grass carp surimi can assist in the preservation of dense and well-connected protein networks during multiple freeze-thaw cycles. These findings are closely aligned with those noted above for Ca^2+^ATPase, total sulfhydryl content and FTIR, where the addition of EGCG-TFO (3%) inhibited the FCY-induced oxidative modifications and prevented the variation in Amide *I* and II spectral regions.

### 3.8 Myosin homology modeling and sequence alignment

**Fig. 7:**
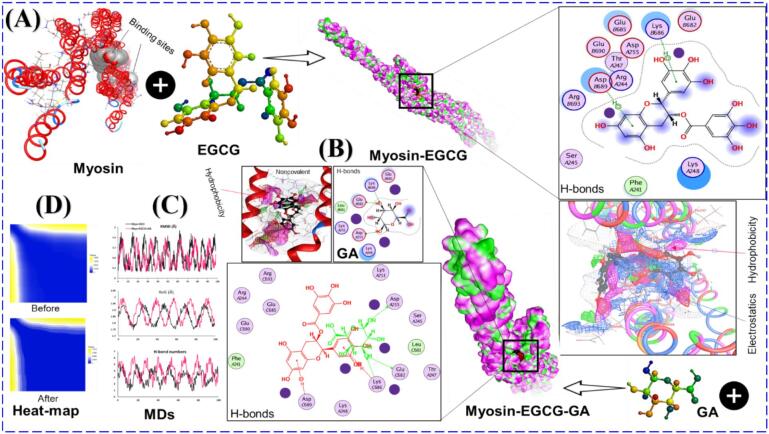
**(A)** 3D representation of myosin template (template point, 1C1G) by homology modeling and its amino acids sequences at 7 A° resolution in spermine-induced crystal form and their three key binding sites. **(B)** 2D-strucutre of each ligand used, interaction between myosin and multiple ligands, 3D-bidning structure, hydrophobic forces, electrostatic binding, final surface conjugate. **(C)** Root means square deviation (RMSD), Radius of gyration (RoG), and H-bond interactions number after 100 ns of simulation. **(D)** Heat-map snaps.

The objective of sequence alignment and homology modeling is to further understand the interaction between tea flower oligosaccharide-based fraction galactose (GA) and EGCG complexes and the functional regions of myosin. Sequence alignment is still one of the most important steps in homology modeling, as the minimum similarity requirement is typically greater than 40% (greater than 50% provides greater confidence in docking and structural validity). Our results indicated that our target myosin sequence demonstrated 54% similarity to our selected template (PDB ID:1C1G) and therefore we decided to utilize this template for myosin homology modeling (Fig. 7a). As a consequence of not having a complete crystal structure for myosin available for use, we decided to perform homology modeling to develop a reliable 3D structure. The myosin heavy chain is involved in actin binding, ATPase activity and conformational flexibility (Lin et al., 2024). Consequently, we modeled the myosin heavy chain and used it for docking and molecular dynamic simulations. Our validated myosin model showed that all functional domains were folded properly and displayed a correct spatial orientation. Our final model contained three ligand-accessible binding sites and these locations were represented as targeted receptor sites for EGCG-GA complex docking. The orientation of the myosin model and its binding regions are shown in Fig. 7A.

### 3.9 Molecular docking

Molecular docking simulations were performed on EGCG–GA adducts with myosin, as shown in Fig. 7B. Based on the results of these docking studies, the EGCG–GA adduct had a much greater affinity for myosin than either saccharide alone, indicating a synergistic effect between EGCG and the GA portions of the adduct. The first five poses of the docking simulation, as indicated in Table 2, had binding scores within the −6.85 to −6.21 kcal/mol range, suggesting that the interactions between EGCG-GA and myosin are thermodynamically favorable. Pose 1, which had the lowest binding energy of −6.85 kcal/mol, also had a reasonable RMSD-refine of 2.18 Å, indicating that these two values are representative. The detailed analysis of the interactions showed that the hydroxyl groups on galactose and the phenolic OH groups on EGCG both participated in hydrogen bonding with myosin residues. Hydrogen bonds were formed between EGCG and the backbone and side-chain atoms of Lys (C686), Glu (C682), Asp (A225), and Asp (C689). In addition to GA H-bonds were identified with Lys (B686), Glu (B685), Asp (A255), as well as electrostatic and hydrophobic interactions were recorded between EGCG-GA and myosin (Fig. 7B). The incorporation of EGCG increased the rigidness of GA as a ligand, thereby increasing the density of interaction points and facilitating GA to more firmly anchor itself within the myosin binding pocket. The 2D interaction diagrams (Fig. 7B) that accompany this discussion provide additional support for a cooperative binding mechanism in which EGCG acts as a stabilizing scaffold, with GA providing multiple hydrogen bond donors. This mode of cooperative binding significantly increases the specificity and affinity of binding.

**Table 2:** The top 5 poses of the interaction between myosin and EGCG/TFO and their docking properties.

Number	rseq	mseq	S	rmsd-refine	E_conf	E_place	E_score1	E_refine	E_score2
1	1	1	−6.85	2.18	45.23	−89.45	−12.58	−22.89	−6.85
2	1	1	−6.62	1.89	52.36	−85.21	−11.96	−23.45	−6.62
3	1	1	−6.48	2.45	48.78	−87.65	−12.14	−21.58	−6.48
4	1	1	−6.35	2.01	54.68	−83.47	−11.78	−22.15	−6.35
5	1	1	−6.21	1.65	61.25	−81.23	−11.52	−20.98	−6.21

### 3.10 Molecular dynamic simulation

The stability of the EGCG-GA-myosin complex was determined by means of a molecular dynamic simulation for 80 ns to determine the stability of EGCG was in solution over time. Root Mean Square Deviation (RMSD), radius of gyration (RoG), hydrogen bond occupancy, and hydrophobic surface mapping were used to identify the stability of the EGCG-GA-myosin complex throughout the period. An analysis of the RMSD over time (Fig. 7C) showed a moderate increase during the initial second of equilibrium (20–80 ns) and a return to stable levels (average RMSD 1.95 ± 0.03 Å). This indicates that EGCG-GA forms a stable protein-ligand complex for prolonged periods. Additionally, the RMSD values of EGCG-GA were somewhat greater than those of pure H₂O; however, instead of indicating structural instability, they directed that there were conformational rearrangements due to adaptation to the new, more stable state (Zhai et al., 2024). The RoG values (Fig. 7C) remained fairly static throughout the simulation period, indicating that EGCG-GA maintained the structure of myosin in a more compact and denser conformation. The hydrogen bond analysis (Fig. 7C) was consistent with the structure of the EGCG-GA-myosin complex, which showed that hybridization is consistent with the presence of persistent hydrogen bonds between the hydroxyl groups of GA and myosin, confirming that hybridization plays an important role in maintaining the structural integrity of the complex. The hydrophobicity heatmap (Fig. 7D) shows that the contact of GA with myosin causes a localized distribution of hydrophobic regions on the surface of myosin, specifically around the binding site (green areas), resulting in the reduced exposure of hydrophobic amino acids to water molecules in close proximity. This relocation of hydrophobic residues serves to confirm the proposed cryoprotective function of oligosaccharides derived from tea flowers.

### 3.11 Interaction energies of myosin–H₂O/EGCG–GA complexes

The interaction energy of myosin molecules with solution-containing electrolytes was investigated between 20 and 80 ns, determining the electrostatic interaction energies (Table 3). The contribution of the hydration energies of myosin in H_2_O was due to the large amount of electrostatic interaction −64,251.1 ± 875.6 kJ/mol. The source of this electrostatic interaction is from the extensive hydrogen bond network of water; thus, the concentration of myosin in H_2_O will support high concentrations of myosin, leading to a high hydration energy of myosin-EGCG, which showed a reduction in electrostatic interaction; in contrast, GA showed a greater reduction of the electrostatic interaction energy of myosin – H_2_O as well as the reduction of water molecules due to displacement of water molecules. This displacement of water molecules by GA compensated for the reduction in electrostatic energy by providing stabilization via the direct electrostatic and van der Waals interactions of GA with myosin. A significant contribution of the electrostatic interaction of GA with myosin was 10,000.0 ± 450 (kJ/mol) and of the van der Waals 2600.0 ± 115 kJ/mol were present It should be noted that the total electrostatic energy of the EGCG-GA system was significantly less than that of the H_2_O only system; however, the net interaction energy (myosin-H_2_O + myosin-Ligand) Interaction resulted in an increase in structural stability of the myosin; thus, the benefits of the interactions described previously. Furthermore, GA's ability to displace interfacial water molecules from myosin resulted in decreased flexibility and reduced excess conformational changes in myosin. In addition, the van der Waals interactions of GA with myosin provide further stabilization of the myosin-GA complex by reinforcing these interactions. Thus, the results indicate that electrostatic interactions are still the dominant stabilizing force, but that van der Waals forces contribute to the structural stabilization of myosin. Furthermore, no significant contribution of the mechanisms of vitrification or entrapment was noted, which indicates that the stabilizing mechanisms of myosin are due to preferential interaction and water displacement. The synergistic effect of EGCG and GA stimulates hydrogen bonding, alters hydration dynamics, and enhances myosin heat resistant structural damage.

**Table 3:** Interaction energies (kJ/mol) between myosin and EGCG/TFO molecules in different solutions after MDs.

Interaction energies (kJ/mol)	H₂O-system	EGCG/GA system
Myosin-H₂O		
Van der Waals force	−5114.4 ± 190.2	−4700.0 ± 170
Electrostatic binding	−64,251.1 ± 875.6	−58,000.0 ± 800
Subtotal	−71,051.0 ± 935.5	−62,700.0 ± 850

Myosin-ligands		
Van der Waals force	–	−2600.0 ± 115
Electrostatic binding	–	−10,000.0 ± 450
Subtotal	–	−12,600.0 ± 470

## 4 Conclusion

These results indicate that FCY plays a significant role in the oxidation, denaturation and microstructural alterations of snakehead proteins. FCY accelerated the decrease in Ca^2+^ATPase activity and total sulfhydryl content, as well as the increase in carbonyls and surface hydrophobicity. FCY also affected the structural and microstructural attributes of surimi protein due to oxidation, denaturation and unfolding of functional protein sites. The K samples supplemented with the conventional combination of cryoprotectants (sucrose and sorbitol) prevented oxidative modifications in comparison to EGCG-TFO (1%); however, this stability was not sufficient to mitigate these changes during multiple FCY. Moreover, the 3%-added samples exhibited pronounced abilities against oxidation indicators by establishing a potential hydrogen interaction with amino acids, reducing the amount of water in the protein matrix, as well as ice crystal damage. The addition of EGCG-GA improved the stability by establishing stronger interactions, such as H-bond with Lys (C686), Glu (C682), Asp (A225), Asp (C689), and reduced electrostatic interactions. Moreover, the microstructure of proteins was well-protected by shielding the α-helix, CH bonds and SH groups against potential oxidation threats during 8 FCY. Thus, the current findings establish that EGCG-TFO (3%) is effective in preventing oxidation and related modifications in aquatic proteins. Future studies can be performed at the molecular level to understand the changes in proteins after the incorporation of EGCG-TFO and its effect on the overall sensory attributes of surimi-related products. Moreover, the practicality of EGCG-TFO at the pilot scale can be assessed in terms of extraction cost, application and efficiency on a larger scale to meet industrial needs.

## CRediT authorship contribution statement

**Noman Walayat:** Writing – original draft. **Shufan Ren:** Formal analysis. **Piotr Kulawik:** Writing – review & editing. **Asad Nawaz:** Investigation. **Isam A. Mohamed Ahmed:** Writing – review & editing, Visualization. **Moneera O. Aljobair:** Writing – review & editing. **Ibraheim Khalifa:** Writing – review & editing, Validation. **Zhucheng Su:** Supervision. **Ran Wei:** Supervision, Funding acquisition. **Suleiman A. Althawab:** Writing – review & editing.

## Fundings

This work was supported by funds from Zhejiang Province Key Research and Development Project (2023C04028) provided for Ran Wei. The authors extend their appreciation to the Ulma NAWA Program BNI/ULM/2024/1/00165/U/00001. The authors extend their appreciation to the financial support from Ongoing Research Funding program (ORF-2026-1074), 10.13039/501100002383King Saud University, Riyadh, Saudi Arabia and the Researchers Supporting Project Number (PNURSP2026R251), 10.13039/501100004242Princess Nourah bint Abdulrahman University, Riyadh, Saudi Arabia.

## Declaration of competing interest

The authors declare no conflict of interest.

## Data Availability

Data will be made available on request.

## Appendix A Supplementary data

Supplementary material Image 1
