# Supplementary material for: Synergistic effect of tea epigallocatechin gallate and tea flower-derived oligosaccharides on frozen storage cycles inducing oxidation and microstructural alterations of snakehead surimi proteins
Source: Food Chem X. 2026 Jan 9;33:103518. doi: 10.1016/j.fochx.2026.103518 (PMC12828746; doi:10.1016/j.fochx.2026.103518)
Supplement: Supplementary file 1 — Supplementary material [file mmc1.docx]

**Figure S1:** Preparation of Tea Flower Oligosaccharides


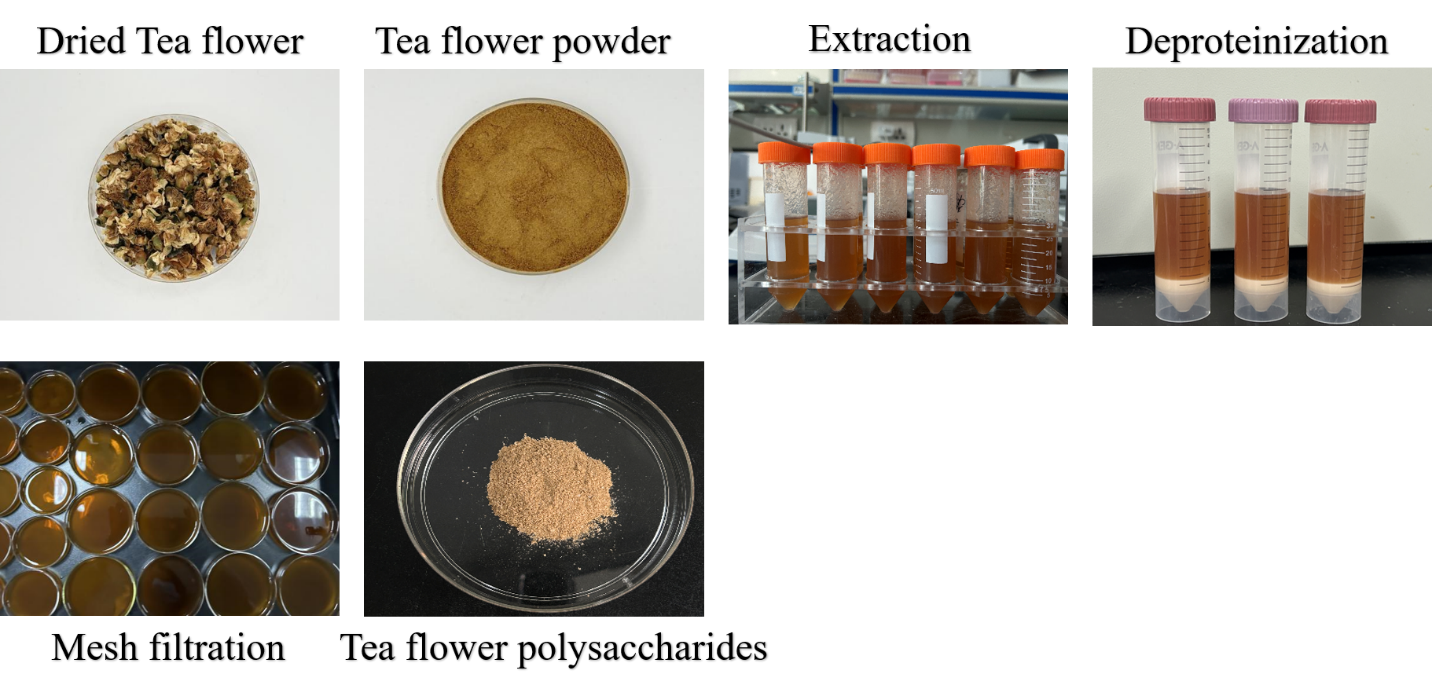


**Table S1:** Investigation on Antioxidant Activity of Oligosaccharide Compound with EGCG

EGCG：TFO ratio: 1:1, 1:2, 2:1

| **Concentration**  **（ug/ml）** | **Reducing Capacity（%）** | | |
| --- | --- | --- | --- |
|  | **2：1** | **1：2** | **1：1** |
| 60 | 5.64±0.04^A^ | 2.33±0.01^A^ | 2.44±0.01^A^ |
| 70 | 6.19±0.05^B^ | 2.60±0.02^B^ | 2.82±0.02^B^ |
| 80 | 6.68±0.06^C^ | 2.87±0.01^C^ | 3.17±0.01^C^ |
| 90 | 7.17±0.07^D^ | 3.11±0.01^D^ | 3.55±0.04^D^ |
| 100 | 7.64±0.05^E^ | 3.37±0.02^E^ | 3.95±0.02^E^ |

Values are expressed as mean ± SD (n = 3). Different superscript letters (A–E) within each column indicate significant differences among concentrations at p < 0.05 according to Tukey’s HSD post hoc test following one-way ANOVA. Increasing letters (A → E) represent increasing Reducing capacity with concentrations.

**Figure S2:** Determination of reducing capacity at different ratios of EGCG-TFO (2:1, 1:2, 1:1)


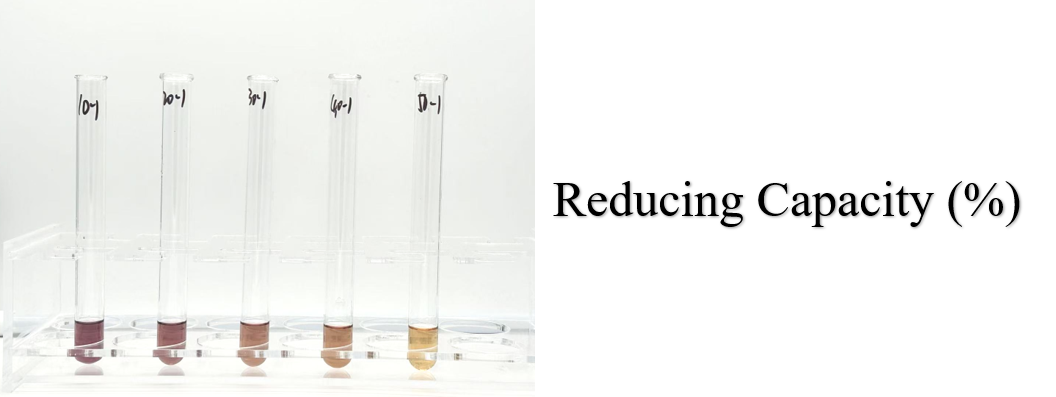


**Table S2:** Investigation on DPPH and ABTS of Oligosaccharide Compound with EGCG

EGCG：TFO ratio: 1:1, 1:2, 2:1

| **Concentration**  **（ug/ml）** | **DPPH** | | **ABTS** | | | |
| --- | --- | --- | --- | --- | --- | --- |
|  | **2：1** | **1：2** | **1：1** | **2：1** | **1：2** | **1：1** |
| 60 | 82.03±0.09^A^ | 13.37±0.39^A^ | 17.64±0.15^A^ | 71.19±0.52^A^ | 19.42±0.19^A^ | 21.9±0.28^A^ |
| 70 | 89.57±0.35^B^ | 21.24±0.64^B^ | 27.72±0.49^B^ | 79.98±0.17^B^ | 26.63±0.68^B^ | 33.88±0.24^B^ |
| 80 | 92.25±0.10^C^ | 26.65±0.05^C^ | 35.14±0.39^C^ | 86.17±0.77^C^ | 34.39±0.56^C^ | 44.17±0.42^C^ |
| 90 | 92.27±0.09^C^ | 34.16±0.59^D^ | 42.93±0.3^D^ | 91.49±0.15^D^ | 42.19±0.40^D^ | 52.48±0.24^D^ |
| 100 | 92.37±0.07^C^ | 34.10±0.49^D^ | 50.45±0.49^E^ | 95.37±0.26^E^ | 49.86±0.95^E^ | 62.40±0.78^E^ |

Values are expressed as mean ± SD (n = 3). Different superscript letters (A–E) within each column indicate significant differences among concentrations at p < 0.05 according to Tukey’s HSD post hoc test following one-way ANOVA. Increasing letters (A to E) represent increasing DPPH and ABTS with concentrations.

**Figure S3:** Determination of DPPH and ABTS at different ratios of EGCG-TFO (2:1, 1:2, 1:1)


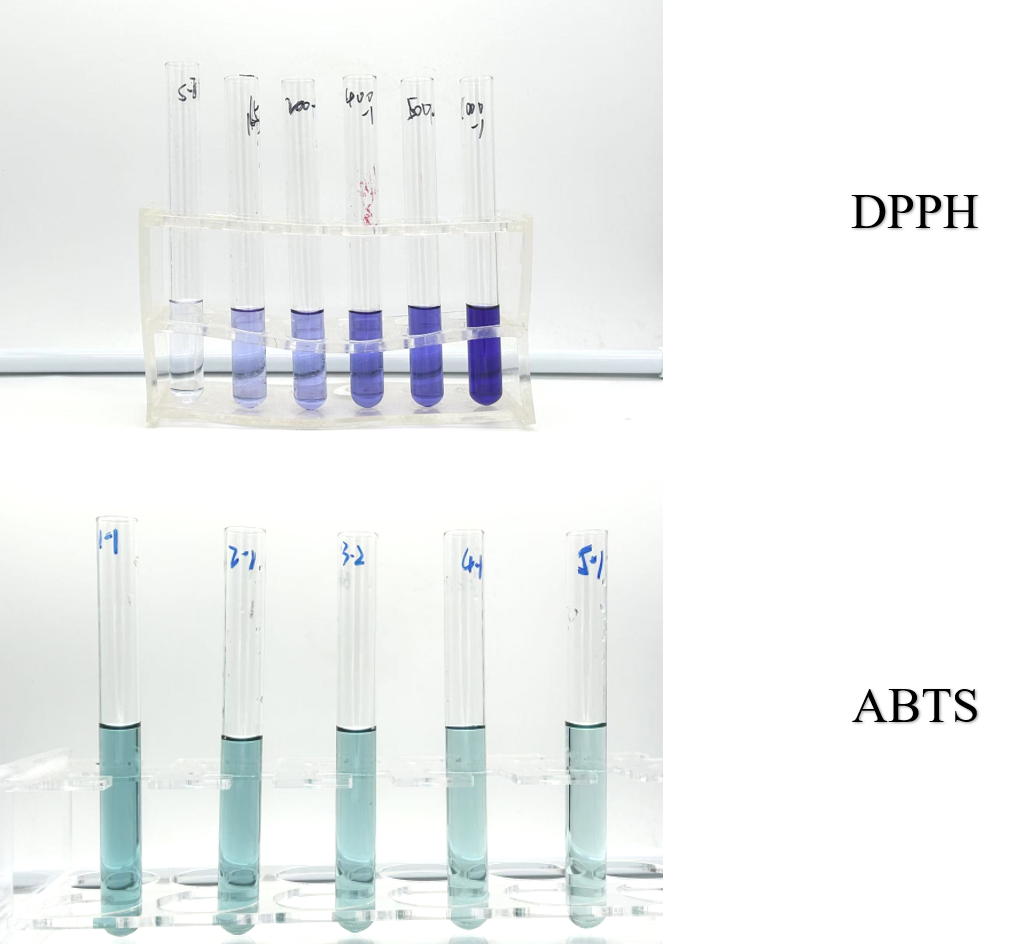


**Supplementary data**

**Table S3.** The statistical analysis output of the measured parameters.

| Time | Treatments | ANOVA (F-value) | | P-value | Effect size (η²) | Levene's test (p-value) |
| --- | --- | --- | --- | --- | --- | --- |
| Ca2+-ATP assay activity | | | | | | |
| 0FCY | **C** | 2.45 | | 0.147 | 0.479 | 0.942 |
|  | **K** |  |  |  |  |  |
|  | **EGCG-TFO (1%)** |  |  |  |  |  |
|  | **EGCG-TFO (3%)** |  |  |  |  |  |
| 2FCY | **C** | 124.56 | | <0.001 | 0.979 | 0.795 |
|  | **K** |  |  |  |  |  |
|  | **EGCG-TFO (1%)** |  |  |  |  |  |
|  | **EGCG-TFO (3%)** |  |  |  |  |  |
| 4FCY | **C** | 156.78 | | <0.001 | 0.983 | 0.835 |
|  | **K** |  |  |  |  |  |
|  | **EGCG-TFO (1%)** |  |  |  |  |  |
|  | **EGCG-TFO (3%)** |  |  |  |  |  |
| 6FCY | **C** | 89.34 | | <0.001 | 0.971 | 0.721 |
|  | **K** |  |  |  |  |  |
|  | **EGCG-TFO (1%)** |  |  |  |  |  |
|  | **EGCG-TFO (3%)** |  |  |  |  |  |
| 8FCY | **C** | 67.89 | | <0.001 | 0.962 | 0.757 |
|  | **K** |  |  |  |  |  |
|  | **EGCG-TFO (1%)** |  |  |  |  |  |
|  | **EGCG-TFO (3%)** |  |  |  |  |  |
| Carbonyl contents | | | | | | |
| 0FCY | **C** | 45.23 | | <0.001 | 0.944 | 0.741 |
|  | **K** |  |  |  |  |  |
|  | **EGCG-TFO (1%)** |  |  |  |  |  |
|  | **EGCG-TFO (3%)** |  |  |  |  |  |
| 2FCY | **C** | 156.78 | | <0.001 | 0.983 | 0.685 |
|  | **K** |  |  |  |  |  |
|  | **EGCG-TFO (1%)** |  |  |  |  |  |
|  | **EGCG-TFO (3%)** |  |  |  |  |  |
| 4FCY | **C** | 289.45 | | <0.001 | 0.991 | 0.765 |
|  | **K** |  |  |  |  |  |
|  | **EGCG-TFO (1%)** |  |  |  |  |  |
|  | **EGCG-TFO (3%)** |  |  |  |  |  |
| 6FCY | **C** | 512.34 | | <0.001 | 0.995 | 0.826 |
|  | **K** |  |  |  |  |  |
|  | **EGCG-TFO (1%)** |  |  |  |  |  |
|  | **EGCG-TFO (3%)** |  |  |  |  |  |
| 8FCY | **C** | 678.92 | | <0.001 | 0.996 | 0.725 |
|  | **K** |  |  |  |  |  |
|  | **EGCG-TFO (1%)** |  |  |  |  |  |
|  | **EGCG-TFO (3%)** |  |  |  |  |  |
| Surface hydrophobicity | | | | | | |
| 0FCY | **C** | 0.285 | | 0.836 | 0.096 | 0.924 |
|  | **K** |  |  |  |  |  |
|  | **EGCG-TFO (1%)** |  |  |  |  |  |
|  | **EGCG-TFO (3%)** |  |  |  |  |  |
| 2FCY | **C** | 8.67 | | 0.009 | 0.765 | 0.864 |
|  | **K** |  |  |  |  |  |
|  | **EGCG-TFO (1%)** |  |  |  |  |  |
|  | **EGCG-TFO (3%)** |  |  |  |  |  |
| 4FCY | **C** | 42.78 | | <0.001 | 0.941 | 0.806 |
|  | **K** |  |  |  |  |  |
|  | **EGCG-TFO (1%)** |  |  |  |  |  |
|  | **EGCG-TFO (3%)** |  |  |  |  |  |
| 6FCY | **C** | 78.92 | | <0.001 | 0.967 | 0.748 |
|  | **K** |  |  |  |  |  |
|  | **EGCG-TFO (1%)** |  |  |  |  |  |
|  | **EGCG-TFO (3%)** |  |  |  |  |  |
| 8FCY | **C** | 95.34 | | <0.001 | 0.973 | 0.766 |
|  | **K** |  |  |  |  |  |
|  | **EGCG-TFO (1%)** |  |  |  |  |  |
|  | **EGCG-TFO (3%)** |  |  |  |  |  |
| Surface hydrophobicity | | | | | | |
| 0FCY | **C** | | 0.892 | 0.489 | 0.250 | 0.365 |
|  | **K** | |  |  |  |  |
|  | **EGCG-TFO (1%)** | |  |  |  |  |
|  | **EGCG-TFO (3%)** | |  |  |  |  |
| 2FCY | **C** | | 12.45 | 0.003 | 0.824 | 0.589 |
|  | **K** | |  |  |  |  |
|  | **EGCG-TFO (1%)** | |  |  |  |  |
|  | **EGCG-TFO (3%)** | |  |  |  |  |
| 4FCY | **C** | | 156.34 | <0.001 | 0.983 | 0.225 |
|  | **K** | |  |  |  |  |
|  | **EGCG-TFO (1%)** | |  |  |  |  |
|  | **EGCG-TFO (3%)** | |  |  |  |  |
| 6FCY | **C** | | 189.67 | <0.001 | 0.986 | 0.178 |
|  | **K** | |  |  |  |  |
|  | **EGCG-TFO (1%)** | |  |  |  |  |
|  | **EGCG-TFO (3%)** | |  |  |  |  |
| 8FCY | **C** | | 98.45 | <0.001 | 0.974 | 0.086 |
|  | **K** | |  |  |  |  |
|  | **EGCG-TFO (1%)** | |  |  |  |  |
|  | **EGCG-TFO (3%)** | |  |  |  |  |

n = 3 replicates per treatment.

Prior to ANOVA, the assumptions of normality and homogeneity of variance were verified using Shapiro-Wilk test and Levene's test, respectively. All data for the four measured parameters (carbonyl content, Ca²⁺-ATPase activity, surface hydrophobicity, and total sulfhydryl content) met the assumptions for parametric analysis (Shapiro-Wilk test: p > 0.05 for all groups across all parameters). Levene's test confirmed homogeneity of variance for carbonyl content (p = 0.741, 0.685, 0.765, 0.826, and 0.725), Ca²⁺-ATPase activity (p = 0.942, 0.795, 0.835, 0.721, and 0.757), surface hydrophobicity (p = 0.924, 0.864, 0.806, 0.748, and 0.766), and total sulfhydryl content (p = 0.365, 0.589, 0.225, 0.178, and 0.086) at 0FCY, 2FCY, 4FCY, 6FCY, and 8FCY, respectively. One-way ANOVA results varied by parameter: for carbonyl content, highly significant differences were observed at all time points (0FCY: F = 45.23, p < 0.001, η² = 0.944; 2FCY: F = 156.78, p < 0.001, η² = 0.983; 4FCY: F = 289.45, p < 0.001, η² = 0.991; 6FCY: F = 512.34, p < 0.001, η² = 0.995; 8FCY: F = 678.92, p < 0.001, η² = 0.996); for Ca²⁺-ATPase activity, no significant differences were found at 0FCY (F = 2.45, p = 0.147, η² = 0.479), but highly significant differences emerged at subsequent time points (2FCY: F = 124.56, p < 0.001, η² = 0.979; 4FCY: F = 156.78, p < 0.001, η² = 0.983; 6FCY: F = 89.34, p < 0.001, η² = 0.971; 8FCY: F = 67.89, p < 0.001, η² = 0.962); for surface hydrophobicity, no significant differences were observed at 0FCY (F = 0.285, p = 0.836, η² = 0.096), significant differences began to emerge at 2FCY (F = 8.67, p = 0.009, η² = 0.765), with highly significant differences at later time points (4FCY: F = 42.78, p < 0.001, η² = 0.941; 6FCY: F = 78.92, p < 0.001, η² = 0.967; 8FCY: F = 95.34, p < 0.001, η² = 0.973); and for total sulfhydryl content, no significant differences were found at 0FCY (F = 0.892, p = 0.489, η² = 0.250), with significant differences emerging at 2FCY (F = 12.45, p = 0.003, η² = 0.824) and highly significant differences at subsequent time points (4FCY: F = 156.34, p < 0.001, η² = 0.983; 6FCY: F = 189.67, p < 0.001, η² = 0.986; 8FCY: F = 98.45, p < 0.001, η² = 0.974). Large effect sizes (η² > 0.75 for all parameters from 2FCY onwards, and η² > 0.90 for most comparisons) indicated that treatment accounted for the majority of variance in all measured parameters.

**Table S4.** Total sulfhydryl contents of MP treated with C, PK, EGCG-TFO (1%) and EGCG-TFO (3%) during frozen fluctuation cycles.

| **Treatments**  **/FCY** | **C** | **K** | **EGCG (1%)** | **EGCG (3%)** | **TFO (1%)** | **TFO (3%)** | **EGCG-TFO (1%)** | **EGCG-TFO (3%)** |
| --- | --- | --- | --- | --- | --- | --- | --- | --- |
| **0 FCY** | 54.68±0.55^Aa^ | 54.93±0.31^Aa^ | 54.62±0.42^Aa^ | 54.75±0.36^Aa^ | 54.58±0.39^Aa^ | 54.62±0.41^Aa^ | 54.49±0.08^Aa^ | 54.68±0.78^Aa^ |
| **2 FCY** | 52.04±0.46^Ab^ | 53.55±0.52^Bb^ | 52.34±0.48^Ab^ | 53.05±0.44^Bb^ | 52.18±0.41^Ab^ | 52.45±0.39^Ab^ | 53.47±0.68^Bb^ | 52.4±0.68^Ab^ |
| **4 FCY** | 48.59±0.21^Ac^ | 42.41±0.16^Bc^ | 42.10±0.32^Ac^ | 44.80±0.29^Cc^ | 41.85±0.27^Bc^ | 43.30±0.25^Cc^ | 42.87±0.24^Bc^ | 45.86±0.49^Dc^ |
| **6 FCY** | 44.64±0.26^Ad^ | 39.90±0.65^Bd^ | 37.11±0.38^Cd^ | 41.60±0.31^Dd^ | 36.80±0.34^Ed^ | 37.25±0.30^Cd^ | 38.46±0.56^Fd^ | 43.70±0.10^Gd^ |
| **8 FCY** | 34.70±0.43^Ae^ | 37.16±0.11^Be^ | 34.81±0.40^Ce^ | 37.60±0.33^Be^ | 33.05±0.37^Ce^ | 34.45±0.36^Ce^ | 35.29±0.85^De^ | 39.50±0.22^Fe^ |

Error bars show the standard deviation (SD) of three replicate measurements Upper case letters (A-D) show significant differences (*P < 0.05*) in different treatments within the same freeze-thaw cycle. Lower case letters (a-e) show the individual treatment within the different freeze-thaw cycles

**Table S5.** Ca^2+^ATPase activity of MP treated with C, PK, EGCG-TFO (1%) and EGCG-TFO (3%) during frozen fluctuation cycles.

| **Treatments / FCY** | **C** | **K** | **EGCG (1%)** | **EGCG (3%)** | **TFO (1%)** | **TFO (3%)** | **EGCG-TFO (1%)** | **EGCG-TFO (3%)** |
| --- | --- | --- | --- | --- | --- | --- | --- | --- |
| **0 FCY** | 0.518±0.008^Aa^ | 0.520±0.003^Aa^ | 0.516±0.005^Aa^ | 0.519±0.004^Aa^ | 0.515±0.004^Aa^ | 0.517±0.006^Aa^ | 0.514±0.004^Aa^ | 0.521±0.007^Aa^ |
| **2 FCY** | 0.405±0.006^Ab^ | 0.429±0.002^Bb^ | 0.410±0.005^Ab^ | 0.423±0.004^Bb^ | 0.408±0.006^Ab^ | 0.412±0.005^Ab^ | 0.424±0.007^Bb^ | 0.472±0.003^Cb^ |
| **4 FCY** | 0.366±0.006^Ac^ | 0.375±0.004^Bc^ | 0.379±0.005^Bc^ | 0.383±0.004^Cc^ | 0.367±0.004^Ac^ | 0.370±0.006^Bc^ | 0.364±0.005^Ac^ | 0.434±0.003^Dc^ |
| **6 FCY** | 0.357±0.005^Ad^ | 0.342±0.004^Bd^ | 0.340±0.004^Bd^ | 0.351±0.003^Ad^ | 0.347±0.005^Bd^ | 0.349±0.004^Bd^ | 0.334±0.004^Bd^ | 0.354±0.002^Ad^ |
| **8 FCY** | 0.304±0.003^Ae^ | 0.313±0.006^Be^ | 0.306±0.004^Ae^ | 0.328±0.003^Ce^ | 0.305±0.004^Ae^ | 0.307±0.005^Ae^ | 0.308±0.003^Ae^ | 0.348±0.005^De^ |

Error bars show the standard deviation (SD) of three replicate measurements Upper case letters (A-D) show significant differences (*P < 0.05*) in different treatments within the same freeze-thaw cycle. Lower case letters (a-e) show the individual treatment within the different freeze-thaw cycles

**Table S6.** Carbonyl contents of MP treated with C, PK, EGCG-TFO (1%) and EGCG-TFO (3%) during frozen fluctuation cycles.

| **Treatments / FCY** | | **C** | **K** | | **EGCG (1%)** | **EGCG (3%)** | **TFO (1%)** | **TFO (3%)** | **EGCG-TFO (1%)** | **EGCG-TFO (3%)** |
| --- | --- | --- | --- | --- | --- | --- | --- | --- | --- | --- |
| **0 FCY** | | 2.12±0.17^Aa^ | 2.14±0.14^Aa^ | | 2.13±0.15^Aa^ | 2.14±0.13^Aa^ | 2.16±0.14^Aa^ | 2.12±0.16^Aa^ | 2.17±0.12^Aa^ | 1.56±0.16^Ba^ |
| **2 FCY** | | 4.16±0.14^Ab^ | 3.01±0.12^Bb^ | | 3.85±0.13^Cb^ | 3.20±0.14^Bb^ | 3.95±0.15^Cb^ | 3.70±0.16^Cb^ | 3.77±0.12^Bb^ | 2.44±0.15^Db^ |
| **4 FCY** | | 5.81±0.16^Ac^ | 3.29±0.13^Bb^ | | 4.10±0.14^Cc^ | 3.65±0.12^Bc^ | 4.25±0.15^Cc^ | 4.90±0.13^Bc^ | 5.37±0.11^Bc^ | 3.89±0.12^Bc^ |
| **6 FCY** | | 8.73±0.12^Ad^ | 5.86±0.21^Bc^ | | 6.50±0.18^Cd^ | 5.95±0.17^Bd^ | 6.70±0.19^Cd^ | 6.30±0.16^Cd^ | 6.04±0.20^Cd^ | 4.14±0.14^Dd^ |
| **8 FCY** | 10.50±0.13^Ae^ | | 6.28±0.11^Bd^ | 7.60±0.16^Ce^ | | 6.11±0.14^Be^ | 8.00±0.17^Ce^ | 7.50±0.18^Ce^ | 8.14±0.15^Ce^ | 5.17±0.18^De^ |

Error bars show the standard deviation (SD) of three replicate measurements Upper case letters (A-D) show significant differences (*P < 0.05*) in different treatments within the same freeze-thaw cycle. Lower case letters (a-e) show the individual treatment within the different freeze-thaw cycles

**Table S7.** Surface hydrophobicity of MP treated with C, PK, EGCG-TFO (1%) and EGCG-TFO (3%) during frozen fluctuation cycles.

| **Treatments / FCY** | **C** | **K** | **EGCG (1%)** | **EGCG (3%)** | **TFO (1%)** | **TFO (3%)** | **EGCG-TFO (1%)** | **EGCG- TFO (3%)** |
| --- | --- | --- | --- | --- | --- | --- | --- | --- |
| **0 FCY** | 23.52±0.22^Aa^ | 23.75±0.30^Aa^ | 23.60±0.25^Aa^ | 23.55±0.21^Aa^ | 23.68±0.24^Aa^ | 23.62±0.26^Aa^ | 23.63±0.22^Aa^ | 23.71±0.29^Aa^ |
| **2 FCY** | 27.45±0.65^Ab^ | 27.31±0.25^Ab^ | 27.60±0.40^Ab^ | 26.90±0.35^Bb^ | 27.80±0.45^Ab^ | 27.20±0.38^Ab^ | 27.51±0.34^Ab^ | 25.44±0.24^Bb^ |
| **4 FCY** | 32.67±0.51^Ac^ | 30.79±0.29^Bc^ | 31.80±0.33^Bc^ | 30.95±0.28^Bc^ | 32.10±0.36^Ac^ | 31.60±0.32^Bc^ | 31.33±0.23^Bc^ | 28.15±0.28^Cc^ |
| **6 FCY** | 38.52±0.44^Ad^ | 35.43±0.18^Bd^ | 36.80±0.40^Bd^ | 35.80±0.35^Bd^ | 37.20±0.42^Ad^ | 36.50±0.38^Bd^ | 35.61±0.19^Bd^ | 31.41±0.25^Cd^ |
| **8 FCY** | 44.25±0.31^Ae^ | 40.19±0.15^Be^ | 42.40±0.35^Ce^ | 39.80±0.30^Be^ | 43.00±0.40^Ce^ | 41.50±0.38^Ce^ | 41.88±0.21^Be^ | 34.65±0.25^De^ |

Error bars show the standard deviation (SD) of three replicate measurements Upper case letters (A-D) show significant differences (*P < 0.05*) in different treatments within the same freeze-thaw cycle. Lower case letters (a-e) show the individual treatment within the different freeze-thaw cycle.
